# How frequently do clusters occur in hierarchical clustering analysis? A graph theoretical approach to studying ties in proximity

**DOI:** 10.1186/s13321-016-0114-x

**Published:** 2016-01-25

**Authors:** Wilmer Leal, Eugenio J. Llanos, Guillermo Restrepo, Carlos F. Suárez, Manuel Elkin Patarroyo

**Affiliations:** Fundación Instituto de Inmunología de Colombia (FIDIC), Bogotá, Colombia; Laboratorio de Química Teórica, Universidad de Pamplona, Pamplona, Colombia; Corporación SCIO, Bogotá, Colombia; Bioinformatics Group, Department of Computer Science, Universität Leipzig, Leipzig, Germany; Universidad del Rosario, Bogotá, Colombia; Universidad Nacional de Colombia, Bogotá, Colombia

**Keywords:** Ties in proximity, Cluster stability, Hierarchical cluster analysis (HCA), Dendrogram, Cluster frequency, Molecular descriptor

## Abstract

**Background:**

Hierarchical cluster analysis (HCA) is a widely used classificatory technique in many areas of scientific knowledge. Applications usually yield a dendrogram from an HCA run over a given data set, using a grouping algorithm and a similarity measure. However, even when such parameters are fixed, ties in proximity (i.e. two equidistant clusters from a third one) may produce several different dendrograms, having different possible clustering patterns (different classifications). This situation is usually disregarded and conclusions are based on a single result, leading to questions concerning the permanence of clusters in all the resulting dendrograms; this happens, for example, when using HCA for grouping molecular descriptors to select that less similar ones in QSAR studies.

**Results:**

Representing dendrograms in graph theoretical terms allowed us to introduce four measures of cluster frequency in a canonical way, and use them to calculate cluster frequencies over the set of all possible dendrograms, taking all ties in proximity into account. A toy example of well separated clusters was used, as well as a set of 1666 molecular descriptors calculated for a group of molecules having hepatotoxic activity to show how our functions may be used for studying the effect of ties in HCA analysis. Such functions were not restricted to the tie case; the possibility of using them to derive cluster stability measurements on arbitrary sets of dendrograms having the same leaves is discussed, e.g. dendrograms from variations of HCA parameters. It was found that ties occurred frequently, some yielding tens of thousands of dendrograms, even for small data sets.

**Conclusions:**

Our approach was able to detect trends in clustering patterns by offering a simple way of measuring their frequency, which is often very low. This would imply, that inferences and models based on descriptor classifications (e.g. QSAR) are likely to be biased, thereby requiring an assessment of their reliability. Moreover, any classification of molecular descriptors is likely to be far from unique. Our results highlight the need for evaluating the effect of ties on clustering patterns before classification results can be used accurately.

**Graphical abstract:**
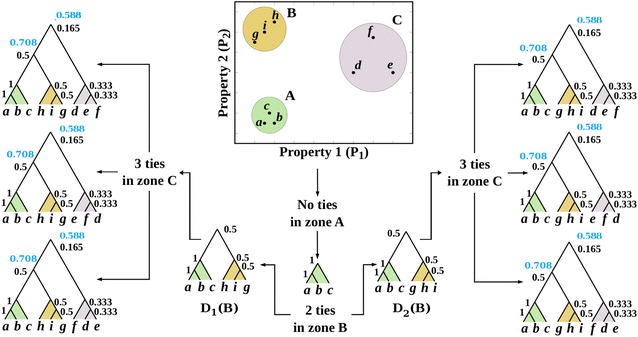
Four cluster contrast functions identifying statistically sound clusters within dendrograms considering ties in proximity

## Background

Classification underlies many scientific enterprises where it provides predictive capability based purely on information about *attributes* of a given set of entities. Classification has been a fundamental step in devising and structuring knowledge in chemistry [1], as illustrated through several classifications of chemicals, e.g. homologous series, chemical elements, amino acids and drugs. It is also a key concept in pattern recognition [2], having broad applications in different fields of knowledge acquisition. In chemo(bio)informatics it is used in different ways [3], e.g. speeding up lead selection in the virtual screening of large databases for chemicals [4], grouping molecules according to structural similarity and biochemical activity in SAR studies [5, 6], or selecting variables in QSAR models [7]. Currently, methods such as *k*-means, hierarchical cluster analysis (HCA) and neural networks (to name but a few) are frequently mentioned in the scientific literature concerning drug design. HCA techniques have also been incorporated into several computational tools for a quite a long time now regarding statistical analysis and are still the subject of many recent computational applications in chemistry [8–10].

According to the ISI Web of Science data, HCA is one of the most used classification methods in chemistry (Aug 17, 2015), being widely used in analytical chemistry, biochemistry, and multidisciplinary chemistry [11]. As a token of this, recent HCA applications are found in drug design [12–14] and in air pollution detection methods [15]. Given the importance of HCA, it must be analyzed and its limitations faced to devise strategies for overcoming them. The aim of this paper is to study some of these shortcomings related to the so called *ties in proximity*.

### HCA and ties in proximity

The objective of HCA is to generate a graph structure (dendrogram) resulting from iterative coupling of clusters according to similarity and grouping criteria. Such a graph structure can also be understood as a collection of neighborhoods leading to a topology, where each cluster becomes in a neighborhood [16]. Any HCA needs a set *X* of elements to classify, a set of attributes $a_i$ characterizing the elements, a similarity function *sf* to quantify resemblance between elements and a grouping methodology *gm* to form clusters of elements [17].

Selecting *sf* and *gm* is usually based on the type of attributes and elements of the set. For instance, the Tanimoto coefficient is the proper *sf* in virtual screening where molecules are represented by fingerprints (attributes); it may be combined with any of Lance and Williams grouping methodologies [18–20]. The final HCA result is a dendrogram (Definition 0.1) depicting a hierarchy of clusters from highest to lowest similarity. Many HCA applications involve an additional step (stopping rule) for pinpointing a similarity value in the hierarchy to select clusters; a review of these rules is found in reference [19].

*X*, $a_i$, *sf*, and *gm* are usually set up in HCA applications and the resulting clusters analyzed. Questions arise regarding the permanence of such clusters when *X*, $a_i$, *sf*, and *gm* are modified or when random noise is added to the input data; the more clusters remain the same, the more reliable the classification is and the results are expected to be inherent to *X* and not artifacts of the HCA method [19]. However, even if *X*, $a_i$, *sf*, and *gm* are set up, the resulting clusters may not always be the same due to *ties in proximity* [21], i.e. equidistances between elements of *X* or between clusters in *X*. A simple tie in proximity results when the similarity between *A* and *B* is the same as that between *B* and *C* ($A, B, C \subset X$), thereby making it troublesome to determine whether *B* is part of a cluster with *A* or with *C*. Ties in proximity are part of the clustering ambiguities that often occur when clustering discrete data (binary, multinomial or count data) or continuous data without sufficient precision [22], which are typical in chemo(bio)informatics [21, 23]. Several clustering algorithms, besides HCA, such as Taylor–Butina leader, Jarvis–Patrick, *k*-means and others [22] treat ties in proximity arbitrarily by making decisions regarding how to break the ties depending on the input order of the data. This arbitrariness leads to ambiguous results that are normally overlooked. More generally, ties in proximity may lead to other ambiguous outcomes within not just HCA, but with most clustering algorithms. Problems concerning the use of discrete data may cascade to ties in merging criterion [22], e.g. Ward’s squared error merging criterion, or the use of performance enhancement routines, e.g. reciprocal nearest neighbors [22]. Even more broadly, other forms of tie breaking can lead to ambiguous results in not only discrete data, but in continuous data, e.g. exclusion region clustering [22].

The ambiguities brought about by ties in proximity depend on several factors related to input data and methodological decisions [22]: (i) size of *X*, the larger the set of elements to classify the more likely the ties; (ii) number of attributes and their precision, typically the lower the number of attributes the more likely there are going to be many ties; the lack of precision may lead to many ties even for cases with many attributes. The number of digits of precision needed to reduce the likelihood of ties is proportional to the size of *X* and the number of attributes. (iii) type of *sf*, for example, for fingerprints, the Euclidean metric is likely to produce a lot more ties than the Tanimoto coefficient and the cosine coefficient, which produces less. For continuous data, the number of ties depends on the number of possible measure values of each $a_i$ and on their distribution, which, depending on the kind of *sf* used, may lead to many ties. (iv) type of *gm*, where groping methodologies that mathematically operate on the data producing new measures, e.g. group average and Ward’s, reduce the number of ties, which contrast with grouping methodologies such as single and complete linkage that operate on the data as they are and that increase the likelihood of ties.

Being aware of clustering ambiguities given by ties in proximity and their aforementioned dependencies, clustering users can make choices to reduce the ambiguities, e.g. by transforming discrete data using binary independent component analysis [24] or they can look for methodological alternatives such as using multidendrograms that group more than two clusters at the same time when ties occur [25], or using pyramidal clustering that allows cluster overlapping [26, 27], or using correlations instead of distances [28] or by exploring the possible solutions and assessing the distances among objects within them [23]. Another possibility is using the Markov chain clustering algorithm [29] that may reduce to a big extent the number of ties, but which depends on the selection of a parameter (inflation factor) that is set up by the researcher. Nevertheless, the probability of ties occurring in large data sets is high [21] and it is therefore imperative to consider the validity of clusters given ties. This paper thus studies how frequently clusters occur in HCA, given a fixed setting up of $X, a_i, sf,$ and *gm*, considering ties in proximity.

## Results and discussion

### Cluster frequency regarding ties in proximity

Our starting point is an HCA algorithm where *X*, $a_i$, *sf*, and *gm* are set up and fixed during the whole HCA study. We devise two extreme situations for exploring cluster frequency given ties in proximity, i.e. tie and no-tie cases. It should be noted that frequency is understood as empirical probability, i.e. the appearance of a cluster in the total number of dendrograms given by ties.

*Tie case* all similarity values between every couple in *X* are the same, including those obtained at each coupling step, i.e. all elements are equidistant.

*No-tie case* all similarity values between every couple in *X* are different, i.e. no equidistance is found during an HCA run.

In the tie case (Proposition 0.1), the frequency for every cluster *C* in the total number of dendrograms given by ties is lower than 1 if *C* has more than two elements (Proposition 0.1, item 2) and clusters having the same number of elements have the same likelihood of showing up (Proposition  0.1, item 3). In the no-tie case, every cluster has frequency 1.

Any HCA result is in between tie and no-tie cases. The question arising is thus how to quantify cluster frequency in *X*, produced by ties, if the distribution of similarity values does not fit the tie or no-tie cases. The following describes our procedure for quantifying such frequencies.

We start by taking a set *X* with *n* elements and run an HCA. As $a_i$, *sf*, and *gm* remain constant, the possible number of dendrograms *F*(*n*) is given by Felsenstein [30].

1 $$\begin{aligned} F(n)=\frac{(2n-3)!}{2^{n-2}(n-2)!} \end{aligned}$$

Since ties may yield $m\le F(n)$ different dendrograms of the possible *F*(*n*), we define $\{D_i\}_{i\le m}$ as the set of such dendrograms. The aim is to determine whether a cluster *C*, derived from *X*, is present in $\{D_i\}_{i\le m}$ and to what extent, this being regarded as the frequency of *C* in the HCA study.

We characterize *C* as a set and as a graph. As a graph, *C* is a subtree (Definition 0.2) of at least one dendrogram in $\{D_i\}_{i\le m}$, i.e. *C* is any *branch* of any of the possible *F*(*n*) dendrograms. As a set, *C* corresponds to the elements of *X* present in the subtree *C*, i.e. $C\subseteq X$.

**Fig. 1:**
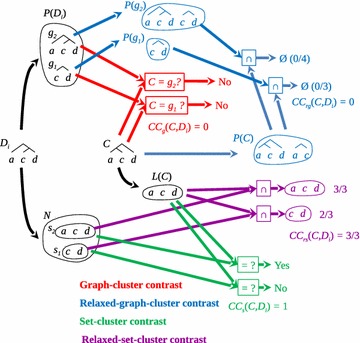
The four contrast functions are schematically depicted by means of an example illustrating how both graph-cluster contrasts yielded different results to those of the set-cluster contrasts. (1) Graph-cluster contrast: $D_i$ is partitioned into its subtrees (*graphs*) $g_1$ and $g_2$, which are gathered in $P(D_i)=\{g_1, g_2\}$. *C* is then contrasted with $P(D_i)$ by assessing whether *C* is one of the elements of $P(D_i)$ (*red*), as in this case it is not, we have $CC_g(C,D_i)=0$. (2) Relaxed-graph-cluster contrast: to quantify the presence of the parts of *P*(*C*) in $P(D_i)$, the parts of $P(D_i)$ are expanded into their respective graph partition sets $P(g_1)$ and $P(g_2)$ (*blue*) determining the common graphs between *P*(*C*) and $P(g_1)$ and between *P*(*C*) and $P(g_2)$, if any. There is no common graph in this example, thus $CC_{rg}(C,D_i)=0$. (3) As set, *C* is characterised by its elements (*leaves*), called $L(C)=\{a,c,d\}$. Graph $D_i$, to be contrasted with *L*(*C*), is characterized by the collection $N=\{s_1,s_2\}$ where $s_j=L(g_j)$ i.e. the set of its leaves. *L*(*C*) is contrasted with *N* by evaluating whether *L*(*C*) is any of the elements of *N* (*green*). In this case, $s_2=L(C)$ and the cluster contrast as set is 1 $CC_s(C,D_i)=1$. (4) *L*(*C*) is intersected with each element of *N* to quantify to what extent the elements of *L*(*C*) are present in the subtrees of $D_i$, yielding sets $\{a,c,d\}$ and $\{c,d\}$ (*purple*). The *first set* indicates that there are three common elements between *L*(*C*) and $\{a,c,d\}$ out of the three elements of *L*(*C*) and $\{a,c,d\}$. The *second set* shows that there are two common elements between *L*(*C*) and $\{c,d\}$ out of the three elements of *L*(*C*) and $\{c,d\}$. It can thus be stated that the cluster contrast of *C* in $D_i$ is 1 (3/3), which is the maximum overlap between *L*(*C*) and *N* (*purple*)

To assess the presence of *C* in $\{D_i\}_{i\le m}$ we select a dendrogram $D_i$ from $\{D_i\}_{i\le m}$ and determine whether *C* is in $D_i$; the same procedure is run over all $D_i$ in $\{D_i\}_{i\le m}$. As *C* is characterized as a graph and as a set, we devise two methods for determining the presence of *C* as a graph and two for determining its presence as a set. We call these methods *cluster contrasts*, which are schematically depicted in Fig. 1.

#### Graph-cluster contrast

We partition $D_i$ into its subtrees (graphs) $g_j$, which are gathered in $P(D_i)=\{g_j\}_{j\in J}$ (Fig. 1). Note that the partitioning is thought of as containing only nontrivial subtrees to avoid the consideration of singletons. *C* is then contrasted with $P(D_i)$ by assessing whether *C* is one of the elements of $P(D_i)$. If that is the case, it is said that the graph-cluster contrast of *C* in $D_i$ is 1, otherwise 0, as is the case in the example of Fig. 1 (red).

The graph-cluster contrast of *C* in $D_i$ is defined as:

2 $$\begin{aligned} CC_g(C,D_i) = {\left\{ \begin{array}{ll} 1 &{} {\text {if }} P(C)=P(g_j),\;\; {\text { for some }} j\in J \\ 0 &{} {\text {otherwise}} \end{array}\right. } \end{aligned}$$

in other words, $CC_g(C,D_i)$ is 1 if and only if *C* is a “branch” of $D_i$ or the whole dendrogram $D_i$.

#### Relaxed-graph-cluster contrast

Our interest then focus on *quantifying* the presence of *C* in $D_i$; this is done by characterizing *C* like $D_i$ in the graph-cluster contrast, i.e. as graph partition set. Hence, the graph partition of *C* is *P*(*C*) (Fig. 1, blue). To quantify the presence of the parts of *P*(*C*) in $P(D_i)$ we expand the parts of $P(D_i)$ into their respective graph partition sets $P(g_1)$ and $P(g_2)$ (Fig. 1, blue). It is against these sets that the graphs of *P*(*C*) are contrasted by intersection, i.e. by determining the common graphs between *P*(*C*) and $P(g_1)$ and between *P*(*C*) and $P(g_2)$. As shown in Fig. 1 (blue), there is no common graph, the contrast is therefore 0 (blue).

The relaxed-graph-cluster contrast of *C* in $D_i$ is defined as:

3 $$\begin{aligned} CC_{rg}(C,D_i)=\max _j\frac{|P(C)\cap P(g_j)|}{|P(C)\cup P(g_j)|} \end{aligned}$$

This cluster contrast is equivalent to calculating the Jaccard index between *P*(*C*) and $P(g_j)$. Current interest regarding contrast lies not only in assessing whether the whole structure of *C* is present in $D_i$, but whether some parts (subdendrograms) of *C* are in $D_i$.

#### Set-cluster contrast

As *C* is characterized as a set too, we devised this cluster-contrast to assess the presence of *C* in $D_i$. Here *C* is characterized by its elements (leaves), which we call the set $L(C)=\{a,c,d\}$. The graph $D_i$, to be contrasted with *L*(*C*), is characterized by its collection of subtrees (their leaves) $N=\{s_j\}_{j\in J}$ (Fig. 1). *L*(*C*) is contrasted with *N* by evaluating whether *L*(*C*) is any of the elements of *N*. If this happens, the cluster contrast is 1, otherwise 0. As shown in Fig. 1 (green), the cluster contrast is 1.

The set-cluster contrast of *C* in $D_i$ is defined as:

4 $$\begin{aligned} CC_s(C,D_i) = {\left\{ \begin{array}{ll} 1 &{} {\text {if }} L(C)=s_j,\;\; {\text { for some }} j \\ 0 &{} {\text {otherwise}} \end{array}\right. } \end{aligned}$$

#### Relaxed-set-cluster contrast

We then quantify to what extent the elements of *L*(*C*) are present in the subtrees (sets) of *N* (Fig. 1, purple). This is done by intersecting *L*(*C*) with each subtree of *N*, which yields sets $\{a,c,d\}$ and $\{c,d\}$ (Fig. 1, purple). The first set indicates that there are three common elements between *L*(*C*) and $\{a,c,d\}$ out of the three elements of *L*(*C*) and $\{a,c,d\}$. The second set shows that there are two common elements between *L*(*C*) and $\{c,d\}$ out of the three elements of *L*(*C*) and $\{c,d\}$. It is therefore possible to state that the cluster contrast of *C* in $D_i$ is 1 (3/3), which is the maximum overlapping between *L*(*C*) and *N* (Fig. 1, purple).

The relaxed-set-cluster contrast of *C* in $D_i$ is defined as:

5 $$\begin{aligned} CC_{rs}(C,D_i)=\max _j\frac{|L(C)\cap s_j|}{|L(C)\cup s_j|} \end{aligned}$$

Figure 1 shows how both graph-cluster contrasts yield different results than the set-cluster contrasts. In graph theoretical terms, *C* is not present in $D_i$, while in set theoretical ones, it is. This occurs as graph-cluster contrasts are more stringent than set ones, for the formers take the hierarchical structure of *C* and $D_i$ into account while the second ones do not. A relaxed-set-cluster contrast equal to 1 (Fig. 1, purple) shows that *C* shares all its elements with a subtree of $D_i$, namely $\{a,c,d\}$, which implies that the set-cluster contrast is also 1 (Fig. 1, green).

Although *C* and $D_i$ are the same in set theoretical terms, they are not the same regarding their structures, as shown in the graph and graph-relaxed-cluster contrasts, which are equal to 0 (Fig. 1, red and blue).

**Fig. 2:**
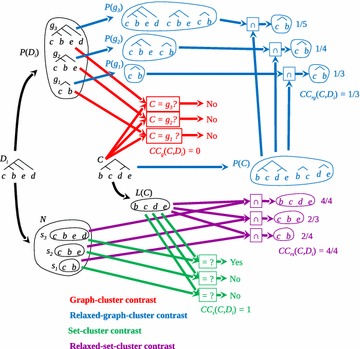
Relaxed graph-cluster contrast $CC_{rg}$ accounts for more details than graph-cluster contrast $CC_g$. In this example *C* is not a subtree of $D_i$, leading to $CC_{g}=0$. Nevertheless, *C* and $D_i$ share a subtree named $g_1$, leading to $CC_{rg}>0$; in fact, they share one out of three subtrees having $CC_{rg}=1/3$. Regarding the set-cluster contrast functions for this example, we have $CC_{s}=1$ meaning a perfect match between the set of leaves of *C* and one of the leaves of one branch (subtree) of $D_i$ i.e. $L(C)=s_3$, implying that $CC_{rs}$ is also 1

**Fig. 3:**
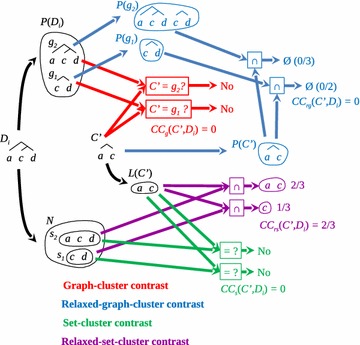
From the aforementioned relationship between set-cluster contrast $CC_s$ and relaxed set-cluster contrast $CC_{rs}$, it can be seen that they differ when the elements of *C* do not coincide exactly with those of any subtree in $D_i$. In this example all cluster contrasts yield a zero value (*red*, *blue*, and *green*), except for $CC_{rs}$ (*purple*). Both elements of *C* are part of the elements of the largest subtree in $D_i$. In fact, both elements of *C* belong to the largest subtree in *N*, $CC_{rs}(C,D_i)$ being equal to 2/3 (*purple*)

To show some other features of each cluster contrast and to analyze the similarities and differences among them, we devise the examples of Figs. 2 and 3.

Figure 2 shows how $CC_{rg}$ accounts for more details than $CC_g$. Whenever we ask for $CC_s$, we wonder whether there is a subtree in $D_i$ such that its elements are the elements of *C*. Hence, $CC_s(C,D_i)$ will always be 1 if and only if the elements of *C* are the elements of a subtree of $D_i$. If $CC_g(C,D_i)=1$, meaning a perfect matching between the graph of *C* and one branch (subtree) of $D_i$. Consequently, a more relaxed matching like that of $CC_{rg}(C,D_i)$ will always be 1 (Proposition 0.3). On the other hand, if $CC_{rg}(C,D_i)=0$, it means that there is no common subtree between *C* and $D_i$, therefore a stringent cluster contrast such as $CC_g(C,D_i)$ (Proposition 0.4) will be 0 (Proposition 0.3).

Another question concerns the following: how many of the subtrees of *C* and $D_i$ are shared? This entails the ratio between the subtrees common to all those considered in the contrast. In the example of Fig. 2, the common subtree is that formed by *b* and *c*, which yields a $CC_{rg}$ of 1/3 (Fig. 2, blue), as the contrast between the subtrees of $P(g_1)$ and *P*(*C*) is what maximizes the ratio between their union and intersection (Eq. 3). As the graph $D_i$ and those derived from *C* do not perfectly match, which gives a non-integer $CC_{rg}$, then the $CC_g$ is 0 (Fig. 2, red).

**Fig. 4:**
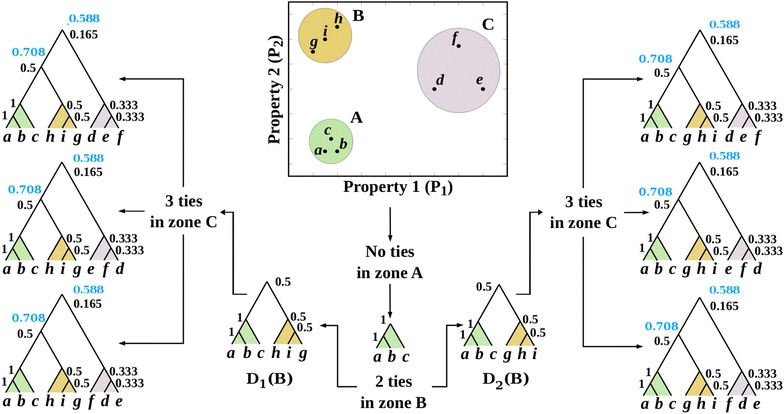
Toy example illustrating how cluster frequency behaves for a data structure having clusters far apart (*A*, *B* and *C*) but having some ties. In this example the set of leaves is $X=\{a,b,c,d,e,f,g,h,i\}$, whose elements are characterised by two properties: $P_1$ and $P_2$. Frequencies of well-separated clusters *A*, *B* and *C* through graph and relaxed-graph-cluster contrasts are depicted (in this example cluster contrast results are equal for graph and relaxed-graph-cluster contrasts)

**Fig. 5:**
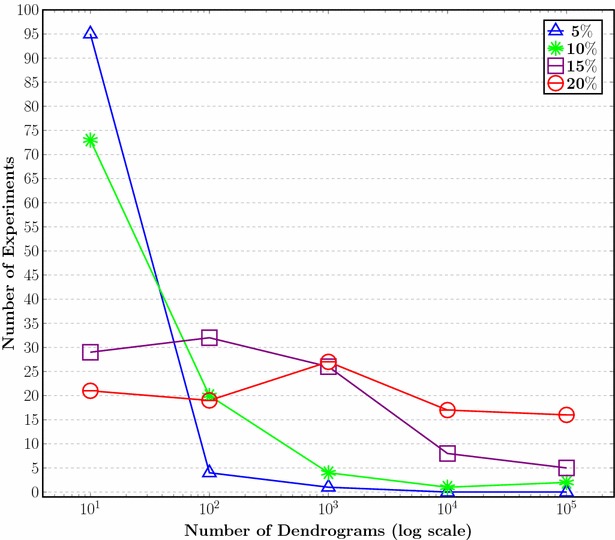
Number of dendrograms resulting from ties in proximity of a classification of molecular descriptors, where 5, 10, 15 and 20 % samples of the original number of descriptors are taken and over which the HCA algorithm is run 100 times

**Fig. 6:**
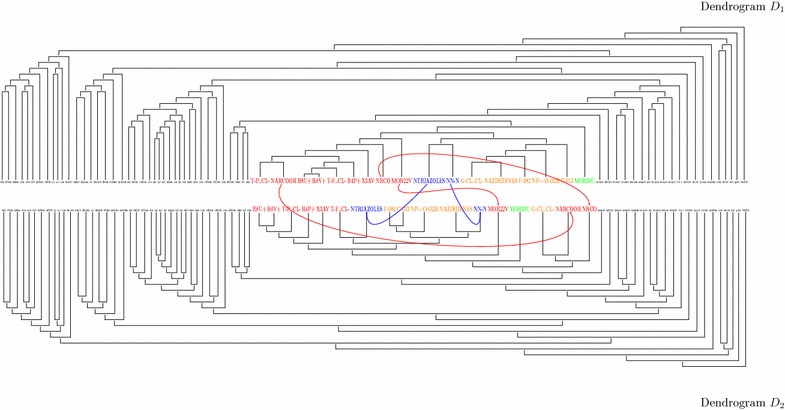
Comparing two dendrograms ($D_1$ and $D_2$) from 5 % samples for the HCA of molecular descriptors shows two conserved zones at *right* and *left-hand sides*, indicating no change in the classification as resulting from ties. The cluster in the center of both dendrograms is divided into four subclusters shown with *different colours*, which change when comparing the two dendrograms. For example, in the *blue cluster*, descriptors NTRIAZOLES and NN-N appear together in $D_1$ and are separated in $D_2$; likewise, it is seen that MOR22V, NARCOOR and NRCO in the red cluster of $D_1$ are together and spread in different clusters in $D_2$

**Fig. 7:**
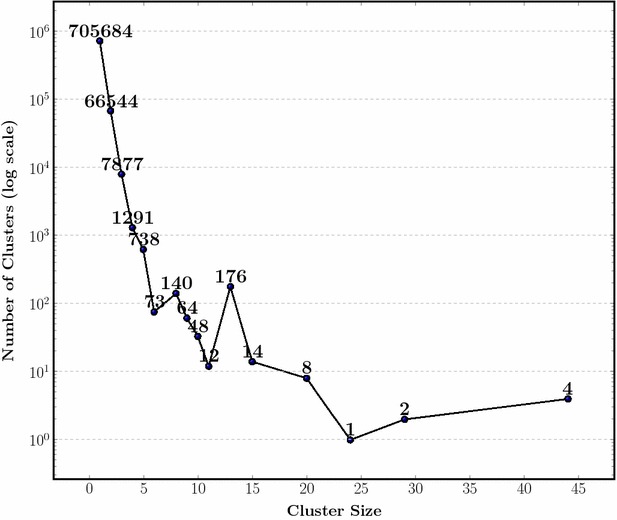
The relationship between cluster size and the frequency of participation in ties. The vast majority of points forming ties (90 % of all ties) are leaves, followed by pairs of leaves. Although the number of ties depends on data distribution, our results showed that the equidistances found in the initial distance matrix might be used for estimating the total number of ties in a given set of objects

**Fig. 8:**
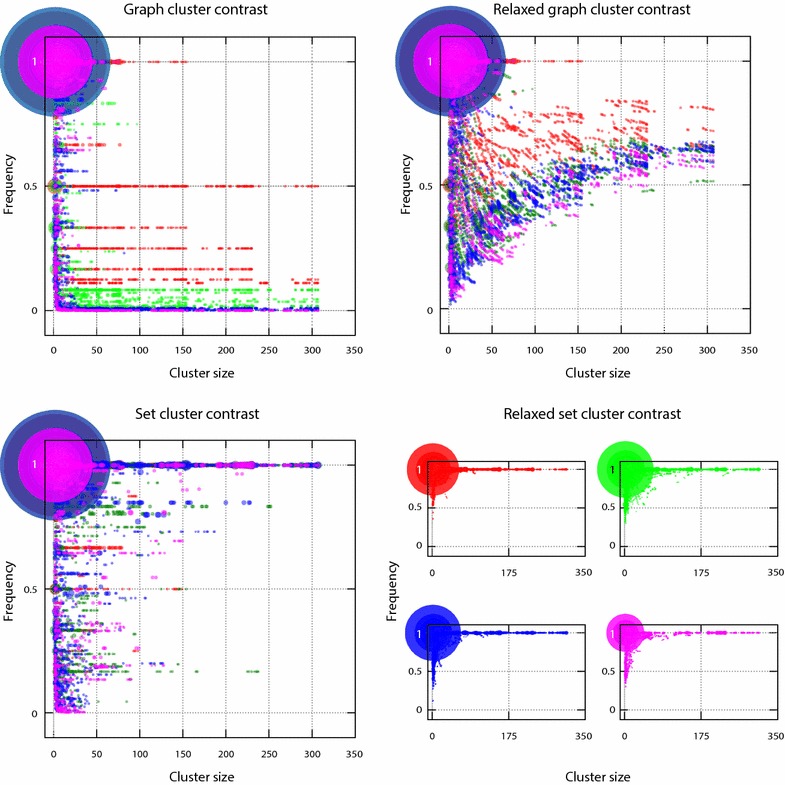
Distribution of cluster frequencies throughout the number of dendrograms, divided into intervals of orders of magnitude: 1–10 (*red*), 10–100 (*green*), 100–1000 (*blue*), $>$1000 (*violet*). Radii of dots are proportional to the number of times the couple cluster size and frequency is found

**Fig. 9:**
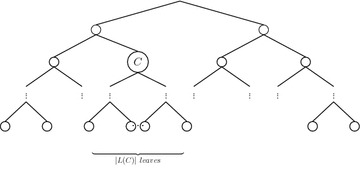
Dendrogram having *C* as a subtree

**Fig. 10:**
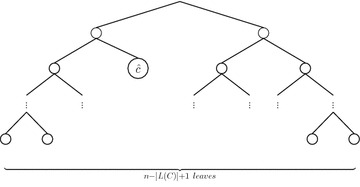
Dendrogram of Fig. 9, where the leave ${\hat{c}}$ represents the cluster *C*

From the aforementioned relationship between $CC_s$ and $CC_{rs}$, we see that they are only different if the elements of *C* are not exactly the same elements of a subtree in $D_i$ (Propositions 0.5 and 0.6). To give an example, we take *C* as shown in Fig. 3, where all cluster contrasts are zero, except for $CC_{rs}$. Thus, when contrasting *C* with $D_i$ (Fig. 3), as graphs, *C* is not a subtree of $D_i$ and the elements of *C* are different from the elements of $D_i$, therefore the cluster contrast of *C* in $D_i$ as a graph and as a set is 0 (Fig. 3, red and green, respectively).

The two elements of *C* are part of the elements of the largest subtree in $D_i$ (Fig. 3). In fact, the elements of *C* (2) are two out of the three of the largest subtree in *N*, which gives a $CC_{rs}(C,D_i)$ of 2/3 (Fig. 3, purple).

### Calculating cluster frequencies assuming ties in proximity

So far, we have shown four methodologies to assess whether a cluster is in a dendrogram, regardless of the actual values of the distances between the elements of the cluster. Recalling our aim, we want to determine the frequency of a cluster in all possible dendrograms obtained by ties. To take them into account, we calculate the frequency of *C*, $f_j (C)$, in $\{D_i\}_{i\le m}$ as a function of the cluster contrast values in each dendrogram $D_i$ with any of the *j*-th cluster contrast methodologies. The frequency is given by:

6 $$\begin{aligned} f_j(C)=\frac{1}{m}\sum _{i=1}^{m}CC_j(C,D_i) \end{aligned}$$

which is an average of the cluster contrasts throughout $\{D_i\}_{i\le m}$. This frequency function allows building stability measures of clusters, as is discussed below.

In general, graph and set cluster contrasts take as the basis for their contrasts the same number of elements. The graph contrast uses subtrees and the set contrast uses sets associated with these subtrees, as there is a one-to-one relationship between subtree and its set representation, the number of elements to contrast in both methods is the same. However, the situation change when referring to the relaxed version of the contrasts. The relaxed graph cluster contrast uses more elements for the contrast than the relaxed set cluster contrast. The relaxed graph requires splitting every subtree (from the cluster and the dendrogram) into subsequent subtrees for further proceed while the relaxed set does any further splitting. Thus, for example in Fig. 1 the relaxed graph uses 5 subtrees (gathered in $P(g_1)$, $P(g_2)$ and *P*(*C*)) while the relaxed set uses 3 subsets. In Fig. 2, the former uses 9 subtrees and the later 4. For dendrograms with many elements such difference is notorious, for the growth of subtrees is faster than that of subsets. This implies that relaxed graph contrast results, for larger sets, yield lower values than relaxed set contrast results. But this is not a problem, for results must be compared in the context of each contrast, i.e. either graph or set. Any conclusion can be drawn from the finding that as relaxed graph the contrast of a cluster in a dendrogram is 0.03 while as a relaxed set the contrast is 0.3.

In practical chemoinformatics applications, set contrasts are of interest for researchers looking for a particular set (cluster) of diverse substances in a compound library that has been previously classified using HCA or for those interested in assessing the validity of a reduced alphabet of amino acids in HCA classification results or for researchers looking for the best partition of a HCA result. For studies where the levels of similarity are important, therefore the hierarchical structure of the dendrograms, the graph contrasts turn important. Cases of these are for example situations where it is known that hundreds of substances are potential candidates for biological screening for antibreast cancer, in this case the graph contrast would look for the different graph structures for those substances, which would indicate which substances can be sent first for screening, namely those which appear in different subtrees of the cluster.

### Examples studied

The different cluster contrasts were applied to two cases: a toy example and a set of molecular descriptors, each one selected to address particular questions. The cluster frequencies were calculated as shown in Eq. 6.

#### Toy example: the frequency of well-separated clusters

This example was designed to show how cluster frequency behaves for a data structure having clusters far apart but having some ties. In this example the set of leaves is $X=\{a,b,c,d,e,f,g,h,i\}$, whose elements are characterized by two properties, as shown in Table 1 (Fig. 4).

**Table 1:** Properties $P_1$ and $P_2$ for elements in *X*

Elements	$P_1$	$P_2$
*a*	1	1
*b*	2	1
*c*	1.5	2
*d*	10	6
*e*	14	6
*f*	12	$6+\sqrt{12}$
*g*	0	9
*h*	2	11
*i*	1	10

The property value $6 + \sqrt{12}$ is used to warranty the equidistance among elements *d*, *e*, and *f*

All four methods for calculating the frequency of *C* work by contrasting it with all possible dendrograms. In this example, all possible dendrograms were built up step-by-step and conclusions drawn regarding cluster frequencies.

- *Cluster frequency through graph-cluster contrast* We explored *X* using the Euclidean metric and the average link method. Initially three clusters were observed: $A=\{a,b,c\}$, $B=\{g,i,h\}$, and $C=\{d,e,f\}$. If the averages of these clusters were ${\bar{x}}$, ${\bar{y}}$ and ${\bar{z}}$, respectively, then it holds the following ordering of distances $d({\bar{x}}, {\bar{y}})<d({\bar{y}}, {\bar{z}})<d({\bar{x}}, {\bar{z}})$, which implies that the calculation of dendrograms considering ties will always lead to the form ((*A*, *B*), *C*). Regarding the internal structure of *A*, *B* and *C*, there was only a possible cluster in *A*, namely ((*a*, *b*), *c*), for $d(a,b)<d(a,c)=d(b,c)$, i.e. $1<1.1=1.1$. Hence, the frequency of (*a*, *b*) was 1, as well as ((*a*, *b*), *c*) (Fig. 4). In *B*, the HCA had two possibilities for starting; it could begin with either (*g*, *i*) or (*h*, *i*); therefore the frequencies of these two clusters were 0.5. If the HCA started with (*g*, *i*), the only remaining clustering possibility being ((*g*, *i*), *h*); this cluster resulted as many times as (*g*, *i*) occured, therefore, the frequency of ((*g*, *i*), *h*) was 0.5. If the HCA algorithm starts with (*h*, *i*), the only remaining clustering possibility was ((*h*, *i*), *g*), as noted before, the frequency was 0.5. For *C*, the elements in it were all equidistant, meeting the requirements of the tie case. Here the HCA algorithm had three possibilities for starting the clustering: (*d*, *e*), (*e*, *f*) and (*f*, *d*), with frequency 0.33. If the algorithm started with (*d*, *e*), it would necessarily group ((*d*, *e*), *f*), implying that the frequency of this cluster was 0.33, for each time (*d*, *e*) showed up, ((*d*, *e*), *f*) appeared too. The other possibilities behaved likewise regarding their frequencies. The next step regarding the HCA algorithm was grouping *A* with *B*. However, *B* had two possible dendrograms, namely $D_1(B)$ and $D_2(B)$ (Fig. 4), therefore the frequency of $D_1(B)$ and $D_2(B)$ was 0.5. The final HCA step is to group *C* with (*A*, *B*). Given one of the two possibilities for (*A*, *B*), there were three possible forms of merging with *C*, for there were three possible dendrograms for *C*. The frequency of having the merging with *C*, given one of the two dendrograms for (*A*, *B*) was $0.5\times 0.33=0.165$. Figure 4 shows all possible clusters in *X* with their respective frequencies, which are shown beside each cluster’s uppermost node. Note that the cluster (*a*, *d*, *g*), for example, has a frequency of 0, for it cannot be found as a graph through the selected HCA algorithm. Likewise, all other clusters not shown in Fig. 4 have 0 frequency when using the HCA algorithm selected and the graph-cluster contrast.
- *Cluster frequency through relaxed-graph-cluster contrast* Here frequency was calculated by considering the number of common subtrees between the cluster and all possible dendrograms. Proposition 0.3 shows that if $CC_{rg}=1$ or 0, then $CC_g=1$ or 0, respectively. Proposition 0.3 stated that clusters having $CC_g=1$ also have $CC_{rg}=1$. Proposition 0.4 shows that these findings implied that $CC_g \le CC_{rg}$. Figure 4 shows the equality case for Proposition 0.4.
  Thence, for the example of well-separated clusters, the frequencies of the clusters depicted in Fig. 4 through the $CC_{rg}$ are shown in blue, where the inequality is shown with values in red and blue.
- *Cluster frequency through set-cluster contrast* Here the graph structure of the cluster and the dendrograms was ignored and, instead, regarded as sets. Due to the isolated nature of *A*, *B* and *C*, we see that each one’s membership of a zone was invariant. This implied that the $CC_s$ of any node having a degree equal to 3 defining a dendrogram with three or more leaves (and of course, the root node) was equal to 1. For Proposition 0.7 the frequency of any cluster having two elements was the same regarding all four frequency functions.
- *Cluster frequency through relaxed-set-cluster contrast* Proposition 0.6 states that if the frequency calculated through $CC_s$ is 1, the frequency based on $CC_{rs}$ is also 1.

#### Frequency of clusters of molecular descriptors characterizing hepatotoxic substances

Molecular descriptors are widely used in modeling substances’ properties and a wealth of descriptors have been developed [31]. A common challenge when modeling a particular endpoint is the selection of relevant descriptors and it has been found that the quality of quantitative structure-activity relationships (QSAR) models depends to a great extent on the type of descriptors used [32].

One of the procedures for such selection is clustering descriptors and the further selection of representative ones within each cluster. An application of the cluster frequency here devised helps in this procedure avoiding ambiguities by ties by looking for those clusters with high frequencies and selecting just in those clusters representative descriptors by traditional methods like the nearest to the centroid of the cluster. These results would contrast with those based on HCA results overlooking ties, which bring a possible classification of many, therefore representative descriptors of not so valid classes of descriptors. As unfortunately, there is no standard descriptor classification as each classification is context-dependent, i.e. a classification of descriptors calculated regarding hydrocarbons yields different results than one with regard to drugs; we devise a classification of molecular descriptors for a particular target of substances. We took substances gathered in the liver toxicity knowledge base (LTKB) containing drugs having the potential to cause drug-induced liver injury (DILI). LTKB is the most authoritative database gathering structural information regarding drugs, as well as DILI annotations for each drug. DILI annotations take into account the causality of hepatotoxicity, the incidence of the liver injury over population and the severity of the damage caused. LTKB assigns one of the following DILI labels to each of the 287 curated drugs: Most-, Less-, and No-DILI-concern.

By discarding drugs which are salts or mixtures and further curation steps [32], the data set was reduced to 273 molecules; 1666 molecular descriptors were calculated using the Internet freeware software E-Dragon, which were then treated with HCA using Euclidean metric and group average linkage, obtaining a ${1666} \times {1666}$ distance matrix upon which cluster frequencies were analyzed.

There were several descriptors having the same values for all molecules (the distance between any pair is zero), and so forming an equivalent class from which one descriptor was chosen; this led to a ${1530} \times {1530}$ distance matrix having a very high likelihood of ties [21], in turn, leading to a combinatorial explosion of dendrograms.

Instead of exploring all dendrograms resulting from ties, we analyzed random samples of the distance matrix accounting for 5, 10, 15, and 20 % of it. Each sample was taken 100 times (experiments). Figure 5 shows the frequency distribution for the amount of different dendrograms per experiment per sample. The distribution of 5 % results shows that most experiments (about 95 %) yielded 1–10 different dendrograms, but there were extreme cases, not so frequent (1 %) where the experiments yielded a thousand dendrograms.

These results contrasted with those for the largest sample (20 %), i.e. 306 descriptors, where the number of different dendrograms were not concentrated on a particular amount of experiments but they were more homogeneously distributed. In fact, 21 and 16 experiments yielded 10 and one hundred thousand dendrograms, respectively.

These results showed that the expected number of different dendrograms increases with sample size, as pointed out by MacCuish [21] and that the ties problem is not only a problem of large data sets, since 5 % of the samples (having only 77 descriptors) had a high likelihood (95 %) of yielding 10 different dendrograms. In fact, one experiment from the 5 % samples produced 132 different dendrograms; we show two of them to illustrate how different they may be (Fig. 6). These results are a matter of concern, for small sets yield different classification results and the problem becomes far much worse for large sets, like those of chemo- and bioinformatic studies, where hundreds of thousands of different results are likely. This makes HCA results, based on a single dendrogram, very unreliable.

Another matter we are interested when exploring ties in a set of molecular descriptors is the cluster size of those subtrees involved in ties, where size is understood as the number of elements (leaves) belonging to the cluster. Figure 7 shows the size of clusters belonging to ties. As tie relationship is at least ternary (one element being equidistant from the other two), most ties involve clusters having single elements (782,676, i.e. 90 % of all clusters involved in ties), followed by clusters having two elements (9 % of all clusters involved in ties). Since the probability of ties increases with the number of elements, the probability of finding a tie decreases throughout the iterations regarding successive cluster couplings. This result highligted the fact that most ties occur at small cluster sizes, where more elements are present in the coupling.

The distribution of cluster sizes formed would be expected to be concentrated around clusters of size two, since single elements are around 90 % of the total number of clusters involved in ties, compared to those of size two which account only for 6 %. Therefore, single elements would most probably be coupled with them than with other sizes. Ties involving clusters of size two and one follow in frequency, and are followed by those of size two coupled with size two. Such trend in the probability of finding a given size involved in ties is not general, because it is possible to design a distribution of points where ties are only found in large sized clusters and no ties in the small ones, particularly on single elements (equidistant centroids at upper stages in the clustering process, far from the leaves). Nevertheless, these cases may be atypical regarding real data sets, and thus a matter of less concern, being a good approach for calculating the number of ties using single elements in real studies (equidistant points in the initial distance matrix).

Although large clusters are not usually involved in ties, they might be affected by the presence of ties of small clusters in a non-trivial manner; this led to exploring how frequency is related to cluster size. For each experiment involving each sample (5, 10, 15, 20 %), which in the end is a distance matrix, all its dendrograms were determined and stored in a file. As mentioned before, some files have a single dendrogram (there are no ties) while others have tens of thousands, which led to 1 and almost negligible frequencies, respectively. The question then arose regarding the distribution of such frequencies, which we explored by splitting the resulting number of dendrograms into intervals of order of magnitude as shown in Fig. 8.

Figure 8 shows that there were many small sized clusters, e.g there were more size two clusters than size 300, and many of them had frequencies lower than those of the other clusters (except for graph-cluster contrast, where there were low frequencies for all cluster sizes); the lower frequencies were for small sized ones. The dot in the upper left-hand corner in each methodology plot shows that there was a great amount of permanent size two clusters.

It was observed that frequencies calculated through set and graph cluster contrasts spread clusters and their frequencies along particular frequency values, which is depicted as series of dots along horizontal lines (Fig. 8). By contrast, frequencies coming from relaxed cluster contrasts were concentrated in frequent small sized clusters (more than 0.5 of frequency) and in very frequent clusters having very different sizes.

Graph-cluster contrast produced frequencies greater than 0.5 just for very small clusters, as there were many ties in the lower levels of the dendrograms (near the leaves). As expected from Proposition 0.4, Fig. 8 shows that graph-cluster contrast yielded lower frequencies than relaxed-graph-cluster contrast. There were many clusters of sizes between 2 and 150, having frequency 1, indicating that such clusters (along with their graph structures) were present in all dendrograms stored in the file.

This indicated that there were clusters gathering 50–150 descriptors that were always grouped together, at least for the molecules from the LTKB database studied here, showing that they were more similar to each other regarding the other descriptors. This may have indicated a large class of descriptors similarly characterizing molecules from the LTKB database. Other results regarding the classification of descriptors, applied to other databases, have given similar results [7].

Regarding the distribution of orders of magnitude, it was observed that clusters in the red zone (showing up in less than 10 dendrograms) had frequencies greater than those in the green zone (appearing in between 10 and 100 dendrograms), the green more than those from the blue zone (showing up in between 100 and 1000 dendrograms) and the blue having more than those in the violet zone (appearing in more that 1000). These results can be explained using Fig. 4: if there were at least two different dendrograms from ties, e.g. the two dendrograms of the first grouping step in Fig. 4, then there would be at least one non-common cluster between them ($\{g,h,i\}$) leading to a frequency of 0.5. Now, if there were three dendrograms, as in the upper right-hand corner of Fig. 4, differing in a single cluster ($\{d,e,f\}$), such a cluster would have a lower frequency (0.333) than that in the previous case. It can be thus concluded that the higher the number of dendrograms coming from ties, the lower the frequency of the non-common clusters.

The relaxed graph cluster-contrast function, unlike graph-cluster one, produces frequencies greater than 0.5 for large clusters. This was because the likelihood of large clusters sharing subgraphs is higher than for small clusters, leading to more overlapping between subgraphs, therefore increasing cluster frequency as relaxed graph. Again, as in graph-cluster contrast frequency, the red zone has greater frequencies than those for the other zones.

For the set-cluster contrast, with a few exceptions, large clusters (more than 100 elements) had high frequencies (greater than 0.75); this was also the case, without exception, for contrast as relaxed set. Indeed, large clusters had frequencies greater than 0.9 for contrast as relaxed set. Unlike graph and relaxed-graph contrasts, often set and relaxed-set contrast frequencies attained a value of 1, the latter case being the most striking one.

### Using the contrast functions as cluster stability measures

Cluster stability is a well-known concept in data analysis, which is intuitively related to the permanence of a cluster throughout a series of *perturbed* results of analyses. This concept is widely used in *k*-means analysis, for example, to select the number of clusters being the one producing the most stable clusters. Stability is measured using a contrast function, such as the Jaccard index, Hamming distance, Rand index or minimal matching distance [33]. Stability is usually calculated by running the algorithm several times, varying some parameters or adding noise to the input data, and then contrasting the *perturbed replicas*.

Our contrast functions do not depend on the distances among elements; in fact, the distance matrix for starting the HCA algorithm may be multiplied by any arbitrary positive factor and the number of ties will remain.

The proposed contrast functions only depend on the elements’ membership of the clusters and their graph structure; this allowed arbitrarily obtained dendrograms to be contrasted only requiring that they share the same set of leaves. An analyst can thus perturb the algorithm or the input data to assess any interesting cluster’s stability. This feature enables designing experiments which test all the sub-patterns in a given dendrogram, using any of the four measures discussed.

## Experimental

A tailormade application for tie detection was developed in Common Lisp (CL) [34], running on *Steel Bank Common Lisp* (SBCL 1.055) under Linux (Ubuntu 14.04), using the *simple average* clustering method. Such application yields (in Newick format [35]) all dendrograms coming from tie detection, generating one file per each input distance matrix. Our utility takes those files as input (the set $\{D_i\}$) along with a file containing clusters (which are sets of graphs), whose frequency is to be calculated through the $CC_j$ contrast functions. The output is a tab separated file containing the input clusters and their frequencies. This utility offers a few options in the command line for controlling the initial parameters for calculations and can be easily incorporated into batch scripts under any Unix-like OS. These dendrograms led to calculating cluster frequencies. Our utility took its input from a file containing clusters which could have been sets or graphs (sub-dendrograms) and from a file having these dendrograms to calculate cluster frequency from the first file. This utility offered a few options in the command line for controlling the initial parameters for calculations and could be easily incorporated into batch scripts under any Unix like OS. The output was a tab separated file containing the input clusters and their frequencies. A few small scripts were written in GAWK 4.0.1 [36], to collect and process the frequency results. These tools are available on request from the authors.

## Conclusions

Even with a fixed HCA methodology, the number of ties in proximity might be very high and depends on the number of elements to classify [21]. The problem of likelihood of clusters throughout a set of dendrograms resulting from HCA, taking into account the vast amount of ties, has been addressed in this paper by proposing four contrast functions, summarized as follows: if one wants to assess cluster frequency in dendrograms resulting from ties, one needs to count the number of times the cluster shows up in these dendrograms. This can be done in several ways, ranging from very stringent approaches to very relaxed ones; from the most to the least stringent approach, one counts the hierarchy of elements (graph-cluster contrast), parts of the hierarchy (relaxed-graph-cluster contrast), only the elements devoid of hierarchy (set-cluster contrast) and parts of the elements (relaxed-set-cluster contrast).

Beside illustrative material (Figs. 1–4), we applied these functions to two data sets, the toy example (a small and illustrative case of nine elements) and a chemo-informatics case (classifying 1666 molecular descriptors). It was found that the likelihood of finding a large amount of different dendrograms was increased along with the number of elements; the toy example yielded six dendrograms (Fig. 4) and the descriptors 115,836 ones. In fact, small data sets (such as 300 elements) may produce between one and one-hundred-thousand dendrograms. This pattern regarding the expected number of dendrograms forces the analyst to determine the frequency of such clusters given the problem of ties in proximity. However, HCA users normally do not do it, thereby making it almost impossible to derive reliable conclusions from a single run of HCA. Essentially, the methods proposed are ways of contrasting sets and graph structures, opening the way forward for applications where the set of dendrograms may be the outcome of situations beyond ties, such as variations on grouping methodologies or on similarity functions or even by adding noise to the input data. In such situations our methods allow for a statistical account of cluster stability. Tie relationship underlies every classificatory distance-based algorithm (it is an equidistance relation) and is very likely to occur. Our contrast functions may easily be generalized to find statistically-sound classes based upon the frequency of their occurrence.

Our results thus showed that classifying molecular descriptors may have been far from unique, taking into account ties in proximity; therefore, the reliability of models based on the classification of descriptors needs to be ascertained as there is a good change of bias. HCA is widely used in chemo- and bioinformatics; the immediate conclusion from this study is thus that if a HCA methodology is used then the conclusions may be very weak, due to the presence of ties. QSAR models in drug design, for instance, strongly depend on the selection of variables, these often being selected using HCA; hence, the usual HCA-based approach for predicting molecular properties through QSAR analysis thus may be based on statistically unsound clusters or simply leaving aside more interesting ones.

## Methods

This section contains a graph theoretical framework for cluster contrasts. Tie and no-tie cases are introduced after Definition 0.1 along with some results on contrast functions $CC_j$ and frequency functions $f_j$. Some general results, whose validity goes beyond tie and no-tie cases are presented. These results are straightforward, but necessary for the validity and optimization of the algorithms and scripts used in this paper. From references [16, 37, 38, 39, 40] we have:

### **Definition 0.1**

A **dendrogram***D* on a set *X* is an acyclic and connected graph having the following kinds of vertices:

1. of degree 1 called **leaves** (elements of *X*),
2. a single vertex of degree 2, called **root node**, and
3. vertices of degree 3, called **nodes**.

### *Remark*

(On the number of clusters within a dendrogram) In reference [41] Restrepo et al. proved that for a set *X* with cardinality *X*, the number of clusters in any of its dendrograms is $2|X|-1$. By removing single clusters the number of remaining clusters is $|X|-1$.

### Tie case

Here all elements in the set *X* are equidistant, i.e. the function $\delta : X \times X \xrightarrow {} {\mathbb {R}}$ used to calculate the dissimilarity between its elements may be defined as:

$$\begin{aligned} \delta (x,y)= {\left\{ \begin{array}{ll} c &{} if \; x \ne y \\ 0 &{} if \; x=y \end{array}\right. } \end{aligned}$$

where *c* is a real number greater or equal to zero. As can be verified, $\delta$ meets the requirements of a metric; therefore $(X,\delta )$ is an **equidistant metric space**, which we use as a representation of the tie case.

In Proposition 0.1 we come up with an expression for the frequency of a cluster *C* regarding the set *X* as an equidistant metric space (tie case), where the frequency is calculated using the graph-cluster contrast. Here $L(C)\subseteq X$ is the set of elements (leaves) of *C*. Before that, *cluster* is defined as a particular kind of sub-graph within a dendrogram according to:

#### **Definition 0.2**

Let *D* be a dendrogram on *X*. A subgraph *C* of *D* is a **subtree (or subdendrogram)** if *C* itself is a dendrogram on *L*(*C*).

#### *Remark*

(On the definition of subtree) An alternative definition of subtree can be found in Restrepo et.al. [16, 37, 38, 39, 40].

#### **Proposition 0.1**

*Let*$(X,\delta )$*be an equidistant metric space and*$C, C^{\prime }$*be clusters such that*$L(C), L(C^{\prime })\subseteq X$*. It follows that:*

1. $$f_g(C)=F(k)/F(n)$$
2. $$|L(C)|\ge 2$$*implies*$$CC_g(C,D_i)<1$$
3. *If*$$|L(C)|=|L(C^{\prime })|$$*, then*$$f_g(C)=f_g(C^{\prime })$$,

*where*$k=n-|L(C)|+1$, *nand* |*L*(*C*)| *are the number of elements inX andC, respectively, andF*(*n*) *is as in Eq. *1.

#### *Proof*

1. Let *F* be the set of dendrograms on *X* considering ties in proximity. As $(X,\delta )$ is an equidistant metric space, then $D_i\in F$ iff $L(D_i)=X$, that is any dendrogram $D_i$ with *X* as set of leaves belongs to *F*. From Eq. 1, we know that there are *F*(*n*) different dendrograms $\{D_i\}_{i\le F(n)}$ on the set *X* of *n* elements, i.e. $F=\{D_i\}_{i\le F(n)}$. In addition, from the definition of cluster frequency function $f_g$ (Eq. 6), we have:
  7 $$\begin{aligned} f_g(C)=\frac{1}{F(n)}\sum _{i=1}^{F(n)}CC_g(C,D_i), \end{aligned}$$
  where $CC_g(C,D_i)=1$ iff *C* is a subtree of $D_i$ and 0 otherwise, which means that $\sum _{i=1}^{F(n)}CC_g(C,D_i)$ is equal to the number of dendrograms from *F* having *C* as a subtree (see Fig. 9) which is equal to the number of dendrograms that can be built on the set $(X\setminus L(C))\cup \{\ {\hat{c}}\}$ where ${\hat{c}}\in L(C)$, i.e. regarding the cluster *C* as a leaf not belonging to $X\setminus L(C)$ (see Fig. 10) As $|X\setminus L(C)\cup \{{\hat{c}}\}|=n-|L(C)|+1$, we have that
  $$\begin{aligned} \sum _{i=1}^{F(n)}CC_g(C,D_i)= F(n-|L(C)|+1), \end{aligned}$$
  by substituting in Eq. 7 with $k=n-|L(C)|+1$, we have (1).
2. $|L(C)|\ge 2$ implies $k=n-|L(C)|+1<n$, which means that
  $$\begin{aligned} CC_g(C,D_i)=\frac{F(k)}{F(n)}<1 \end{aligned}$$
3. It is straightforward since $n-|L(C)|+1=n-|L(C^{\prime })|+1$

$$\square$$

### No-tie case

This occurs when the distance matrix has a unique minimum and, moreover, when each distance matrix keeps having a unique minimum in each HCA step. In such a case, there is only one realizable dendrogram; therefore the frequency of its occurrence as an outcome from the HCA algorithm is 1. The immediate consequence is that the frequency of the cluster in the realizable dendrogram is 1 and the frequency of any other cluster is 0.

### Results beyond tie and no-tie cases

#### **Proposition 0.2**

$CC_{rg}(C,D_k)>0$*iff there is a common graph betweenC, and*$D_k$*whose cardinality is two.*

#### *Proof*

As we do not consider trivial subtrees, then $|C|, |D_k|\ge 2$. Moreover, Eq. 3 leads to $CC_{rg}(C,D_k)>0$ iff $|P(C)\cap P(g_j)|>0$ for some $j\in J$, iff there is a common subgraph between *C* and $g_j$ with two or more elements, as those having one element are disregarded. Therefore, *C* and $g_j$ share a subtree with two elements. $\square$

#### **Proposition 0.3**

8 $$\begin{aligned} CC_{rg}(C,D_k) = {\left\{ \begin{array}{ll} 1 &{} {\text {iff }} CC_g(C,D_k)=1 \\ 0 &{} {\text {if }} CC_g(C,D_k)=0 \end{array}\right. } \end{aligned}$$

#### *Proof*

- If $CC_{rg}(C,D_k)=1$, by Eq. 3,
  9 $$\begin{aligned} CC_{rg}(C,D_i)=\max _j\frac{|P(C)\cap P(g_j)|}{|P(C)\cup P(g_j)|}=1, \end{aligned}$$
  which is equivalent to $|P(C)\cap P(g_j)|=|P(C)\cup P(g_j)|$ for some $j \in J$, iff $P(C)=P(g_j)$ for some $j\in J$. For Eq. 2, it follows that $CC_g(C,D_i)=1$.
- $CC_{rg}(C,D_k)=0$ iff, by Eq. 3,
  10 $$\begin{aligned} \max _j\frac{|P(C)\cap P(g_j)|}{|P(C)\cup P(g_j)|}=0, \end{aligned}$$
  iff $|P(C)\cap P(g_j)|=0$ for all $j\in J$. This is true iff $P(C)\cap P(g_j)= \varnothing$ for all $j \in J$, which implies, according to Eq. 2, that $CC_g(C,D_i)=0$

$$\square$$

#### **Proposition 0.4**

$$CC_g(C)\le CC_{rg}(C)$$.

#### *Proof*

Let $J\subseteq \{1,2,\ldots n\}=I$ be the set of indices such that $CC_g(C,D_j)=1$ for all $j\in J$, then by Proposition 0.3, we have that $CC_{rg}(C,D_j)=1$ for all $j \in J$, therefore:

11 $$\begin{aligned} \sum \limits _{j\in J}CC_g(C,D_j)=\sum \limits _{j\in J}CC_{rg}(C,D_j) \end{aligned}$$

Thus

12 $$\begin{aligned} CC_g(C)= & {} \frac{1}{|I|}\sum \limits _{i\in I}CC_g(C,D_i)\nonumber \\= & {} \frac{1}{|I|}\sum \limits _{j\in J}CC_g(C,D_j)+\frac{1}{|I|}\sum \limits _{i\in I\setminus J}CC_g(C,D_i) \end{aligned}$$

by Eq. 11

13 $$\begin{aligned} CC_g(C)=\frac{1}{|I|}\sum \limits _{j\in J}CC_{rg}(C, D_j)+\frac{1}{|I|}\sum \limits _{i\in I\setminus J}CC_g(C,D_i) \end{aligned}$$

But $CC_g(C,D_i)=0$ for all $i\in I\setminus J$, thus:

14 $$\begin{aligned} CC_g(C)=\frac{1}{|I|}\sum \limits _{j\in J}CC_{rg}(C, D_j). \end{aligned}$$

On the other hand, $CC_{rg}(C,D_i)\ge 0$ for all $i \in I\setminus J$, for if *C* is not in $D_i$ it does not imply that any of the *C* subtrees is in $D_i$. Then

$$\begin{aligned} \sum \limits _{i \in I\setminus J} CC_g(C,D_i)\ge 0 \end{aligned}$$

which implies

15 $$\begin{aligned} CC_g(C)& = \frac{1}{|I|}\sum \limits _{j\in J}CC_{rg}(C, D_j)\nonumber \\ & \le \frac{1}{|I|}\sum \limits _{j\in J}CC_{rg}(C, D_j)+\frac{1}{|I|}\sum \limits _{i\in I\setminus J}CC_{rg}(C,D_i)\nonumber \\ & = {} CC_{rg}(C) \end{aligned}$$

$$\square$$

#### **Proposition 0.5**

$$CC_{s}(C)\le CC_{rs}(C)$$.

#### *Proof*

It follows from a similar argument than the used in Proposition 0.4. $\square$

The following two propositions are important for the performance of the algorithms, for relaxed-set cluster contrasts require more calculations than set- and graph-cluster contrast. On the other hand, Proposition 0.7 is used to avoid extra calculations on the smallest clusters, which, according to our results (Fig. 7) are likely involved in ties.

#### **Proposition 0.6**

$$CC_s(C,D_k)=1 \;\,$$ iff $$CC_{rs}(C,D_k)=1$$.

#### *Proof*

$CC_s(C,D_k)=1$ iff $L(C)= L(g_j)$ for some $j \in J$, where $g_j$ is a subgraph of $D_k$. This is equivalent to

16 $$\begin{aligned} \max _j\frac{|L(C)\cap L(g_j)|}{|L(C)\cup L(g_j)|}=1, \end{aligned}$$

which, by definition, is $CC_{rs}(C,D_k)=1$. $\square$

#### **Proposition 0.7**

*Let Cbe a cluster such that*$|C|=2$*. Then:*

1. If $CC_j(C, D_k)=1$*for some*$j \in \{g, rg, s, rs\}$*, then*$CC_j(C,D_k)=1$*for all*$j \in \{g, rg, s, rs\}$.
2. *If*$CC_j(C, D_k)=0$*for some*$j \in \{g, rg, s, rs\}$*, then*$CC_j(C,D_k)=0$*for all*$j \in \{g, rg, s, rs\}$*.*

#### *Proof*

From Proposition 0.2, $CC_g(C,D_k)=1$ iff $CC_{rg}(C,D_k)=1$ and, from Proposition 0.6, $CC_s(C,D_k)=1$ iff $CC_{rs}(C,D_k)=1$.

Let us prove that $CC_s(C,D_k) = 1$ iff $CC_g(C,D_k)=1$: $CC_s(C,D_k)=1$ is equivalent to $L(C)=L(g_j)$ for some $j \in J$ iff $C=g_j$ for some $j \in J$ (since $|C|=2$), iff $CC_g(C,D_k)=1$.

Moreover, $CC_{rg}(C,D_k) = 1$ iff $CC_g(C,D_k) = 1$ iff $CC_s(C,D_k) = 1$ iff $CC_{rs}(C,D_k) = 1$, which implies that $CC_{rg}(C,D_k) = 1$ iff $CC_{rs}(C,D_k) = 1$

$$\square$$

## References

[1] Schummer J (1998). The chemical core of chemistry I: a conceptual approach. HYLE Int J Philos Chem.

[2] Theodoridis S, Koutroumbas K (2009). Pattern recognition.

[3] Downs GM, Barnard JM (2002). Clustering methods and their uses in computational chemistry. Rev Comput Chem.

[4] Plewczynski D, Spieser SA, Koch U (2006). Assessing different classification methods for virtual screening. J Chem Inf Model.

[5] Kim S, Han L, Yu B, Hähnke VD, Bolton EE, Bryant SH (2015). PubChem structure–activity relationship (SAR) clusters. J Cheminform.

[6] Saeed F, Salim N, Abdo A (2012). Voting-based consensus clustering for combining multiple clusterings of chemical structures. J Cheminform.

[7] Basak S, Niemi G, Veith G (1991). Predicting properties of molecules using graph invariants. J Math Chem.

[8] Gütlein M, Karwath A, Kramer S (2012). CheS-Mapper—chemical space mapping and visualization in 3D. J Cheminform.

[9] Škuta C, Bartůněk P, Svozil D (2014). InCHlib—interactive cluster heatmap for web applications. J Cheminform.

[10] Gobbi A, Giannetti A, Chen H, Lee ML (2015). Atom–atom–ath similarity and sphere exclusion clustering: tools for prioritizing fragment hits. J Cheminform.

[11] Amari S, Aizawa M, Zhang J, Fukuzawa K, Mochizuki Y, Iwasawa Y, Nakata K, Chuman H, Nakano T (2006). VISCANA: visualized cluster analysis of protein–ligand interaction based on the ab initio fragment molecular orbital method for virtual ligand screening. J Chem Inf Model.

[12] Akerman KJ, Fagenson AM, Cyril V, Akerman MP, Munro OQ (2014). Gold(III) macrocycles: nucleotide-specific unconventional catalytic inhibitors of human topoisomerase I. J Am Chem Soc.

[13] Santos-Filho O, Cherkasov A (2008). Using molecular docking, 3D-QSAR, and cluster analysis for screening structurally diverse data sets of pharmacological interest. J Chem Inf Model.

[14] Bellera CL, Balcazar DE, Alberca L, Labriola CA, Talevi A, Carrillo C (2013). Application of computer-aided drug repurposing in the search of new cruzipain inhibitors: discovery of amiodarone and bromocriptine inhibitory effects. J Chem Inf Model.

[15] Lin H, Jang M, Suslick KS (2011). Preoxidation for colorimetric sensor array detection of VOCs. J Am Chem Soc.

[16] Mesa H, Restrepo G (2008). On dendrograms and topologies. MATCH Commun Math Comput Chem.

[17] Bailey KD (1994) Typologies and taxonomies: an introduction to classification techniques. Sage publications, Inc., Thousand Oaks, pp 34–63 [Lewin-Beck M (series editor): Sage University paper series on quantitative applications in the social sciences, vol 102]

[18] Lance GN, Williams WT (1967). A general theory of classificatory sorting strategies: 1. Hierarchical systems. Comput J.

[19] Everitt BS, Landau S, Leese M, Stahl D (2011). Cluster analysis.

[20] Aldenderfer MS, Blashfield RK (1984). Cluster analysis.

[21] MacCuish J, Nicolaou C, MacCuish NE (2001). Ties in proximity and clustering compounds. J Chem Inf Comput Sci.

[22] MacCuish J, MacCuish NE (2011) Clustering in bioinformatics and drug discovery. CRC Press, Boca Ratón (Chapman & Hall: Series on Mathematical and Computational Biology)

[23] Arnau V, Mars S, Marin I (2005). Iterative cluster analysis of protein interaction data. Bioinformatics.

[24] Himberg J, Hyvärine A (2001) Independent component analysis for binary data: An experimental study. In: Lee TW, Jung TP, Makeig S, Sejnowsky TJ (eds) Proceedings of the international workshop on independent component analysis and blind signal separation (ICA2001), pp 552–556

[25] Fernandez A, Gomez S (2008). Solving non-uniqueness in agglomerative hierarchical clustering using multidendrograms. J. Classif.

[26] Bertrand P (1995) Structural properties of pyramidal clustering. In: Cox I, Hansen P, Julesz B (eds) Partitioning data sets. American Mathematical Society, Providence, pp 35–53 (DIMACS Series in Discrete Mathematics and Theoretical Computer Science, vol 19.)

[27] Nicolaou C, MacCuish J, Tamura S (2000) A new multi-domain clustering algorithm for lead discovery that exploits ties in proximities. In: Proceedings from the 13th European symposium on quantitative structure–activity relationships. Prous Science, Barcelona pp 486–495

[28] Prinz S, Avila-Campillo I, Aldridge C, Srinivasan A, Dimitrov K, Siegel AF, Galitski T (2004). Control of yeast filamentous-form growth by modules in an integrated molecular network. Genome Res..

[29] Clustering Ambiguity II. http://learningandotherthings.blogspot.de/2015/07/clustering-ambiguity-ii.html

[30] Felsenstein J (1978). The number of evolutionary trees. Syst. Zool..

[31] Todeschini R, Consonni V (2009). Molecular descriptors for chemoinformatics, volume I: alphabetical listing.

[32] Fourches D, Muratov E, Tropsha A (2010). Trust, but verify: on the importance of chemical structure curation in cheminformatics and QSAR modeling research. J Chem Inf Model.

[33] von Luxburg U (2009). Clustering stability: an overview. Found Trends Mach Learn.

[34] Graham P (1996). ANSI common Lisp.

[35] Felsestein J (2004). Inferring phylogenies.

[36] Robbins A (2001). Effective awk programming.

[37] Restrepo G, Mesa H, Llanos E, Villaveces JL (2004). Topological study of the periodic system. J Chem Inf Comput Sci.

[38] Restrepo G, Mesa H, Llanos E, Villaveces JL, King RB, Rouvray D (2006). Topological study of the periodic system. The mathematics of the periodic table.

[39] Restrepo G, Mesa H, Villaveces JL (2006). On the topological sense of chemical sets. J Math Chem.

[40] Leal W, Restrepo G, Bernal A (2012). A network study of chemical elements: From binary compounds to chemical trends. MATCH Commun Math Comput Chem.

[41] Restrepo G, Mesa H, Llanos E (2007). Three dissimilarity measures to contrast dendrograms. J Chem Inf Comput Sci.

